# Special Issue “Research and Development of Building Materials Based on Industrial Waste”

**DOI:** 10.3390/ma16155231

**Published:** 2023-07-25

**Authors:** Vojtěch Václavík

**Affiliations:** Department of Environmental Engineering, Faculty of Mining and Geology, VSB–Technical University of Ostrava, 17. listopadu 15/2172, 708 00 Ostrava, Czech Republic; vojtech.vaclavik@vsb.cz

This Special Issue, titled “Research and Development of Building Materials Based on Industrial Waste”, is focused on the preparation and description of the properties of new building materials based on industrial waste that are to be used in practice in civil engineering and engineering construction. Recycling and the recovery of by-products and waste facilitate the conservation of primary raw materials and energy resources, mitigate the depletion of natural resources and contribute to the protection of the environment, which is threatened by the accumulation of waste, high dust concentrations, the contamination of watercourses and the appropriation of land.

This Special Issue presents original research results in the following areas: the use of solar panels after the end of their life cycles in the production of cement composites [1]; the radioactivity of building materials in historical buildings [2]; the use of silicon nitride (Si_3_N_4_) and silicon powder (Si) in the production of briquettes with defined properties [3]; the utilization of by-products of solid waste incineration in concrete production [4]; the comparison of the thermal conductivity of aerated concrete based on fly ash [5]; the utilization of by-products of the incineration of solid municipal waste in the production of foam concrete [6]; the mechanical properties of micropowder cement mortar and engineered cementitious composites (ECC), involving investigations of the use of different methods of processing for municipal solid waste incineration (MSWI) as a mineral admixture [7]; the use of marble dust and blast furnace slag in the production of self-compacting concrete [8]; a new method for the synthesis of insulating aerogel via recycling solid waste coal gangue, which has the potential to reduce the industrial production costs of silica aerogels and realize the high-value-added utilization of solid waste [9]; strength tests and numerical simulations of the Loess modified by desulfurization ash and fly ash [10]; research on the rheological properties of cement paste prepared using silica fume [11]; and, finally, research on the properties of cement paste prepared from superfine basalt powder [12].
